# Combination of shear-wave elastography and color Doppler: Feasible method to avoid unnecessary breast excision of fibroepithelial lesions diagnosed by core needle biopsy

**DOI:** 10.1371/journal.pone.0175380

**Published:** 2017-05-04

**Authors:** Ga Ram Kim, Ji Soo Choi, Boo-Kyung Han, Eun Young Ko, Eun Sook Ko, Soo Yeon Hahn

**Affiliations:** 1 Department of Radiology, Samsung Medical Center, Sungkyunkwan University School of Medicine, Seoul, Republic of Korea; 2 Department of Radiology, Inha University Hospital, Inha University School of Medicine, Incheon, Republic of Korea; Rensselaer Polytechnic Institute, UNITED STATES

## Abstract

**Background:**

We evaluated shear-wave elastography (SWE) and color Doppler ultrasonography (US) features for fibroepithelial lesions (FELs), and to evaluate their utility to differentiate fibroadenomas (FAs) and phyllodes tumors (PTs).

**Methods:**

This retrospective study included 67 FELs pathologically confirmed (49 FAs, 18 PTs). B-mode US, SWE and color Doppler US were performed for each lesion. Mean elasticity (E_mean_), maximum elasticity (E_max_), and vascularity were determined by SWE and Doppler US. Diagnostic performances were calculated to differentiate FAs and PTs. Equivocal FELs diagnosed by core needle biopsy (CNB) were further analyzed.

**Results:**

Median E_mean_ and E_max_ were significantly lower for FAs than PTs (E_mean_, 15.7 vs. 66.7 kPa; E_max_, 21.0 vs. 76.7 kPa, P<0.01). Low vascularity (0–1 vessel flow) on color Doppler US were more frequent in FAs than in PTs (*P*<0.01). SWE showed significantly higher specificities (E_mean_ >43.9 kPa, 89.8%; E_max_ >46.1 kPa, 79.6%) than B-mode US (42.9%) (*P*<0.01) for differentiating PTs from FAs. Other diagnostic values of SWE and overall diagnostic values of Doppler US were not significantly different from B-mode US (*P*>0.05). The combination of SWE and Doppler US with ‘E_mean_>43.9 kPa or high vascularity (≥2 vessel flows)’ showed a higher area under the curve (0.786 vs. 0.687) and higher diagnostic values than B-mode US (sensitivity, 100 vs. 94.4%; specificity, 57.1 vs. 42.9%; positive predictive value, 46.2 vs. 37.8%; negative predictive value, 100 vs. 95.5%), without statistical significance (*P*>0.05). Of the 30 equivocal FELs, all lesions with ‘E_mean_≤43.9 kPa and low vascularity (0–1 vessel flow)’ (23.3%, 7/30) were finally confirmed as FAs by excision.

**Conclusion:**

FAs have a tendency to have less stiffness and lower vascularity than PTs. Combined SWE and color Doppler US may help patients with equivocal FELs diagnosed by CNB avoid unnecessary excision.

## Introduction

Fibroepithelial lesion (FEL) of the breast, one of the most common lesions diagnosed by core needle biopsy (CNB), includes fibroadenomas (FAs) and phyllodes tumors (PTs) [1–3]. The majority of FELs are FAs whereas PTs are rare and account for less than 1% of all breast tumors [4]. FAs generally remain in situ and some spontaneously regress, therefore FAs diagnosed by CNB with imaging pathologic concordance can be followed-up with nonoperative management [5,6]. In comparison, PTs can be benign, borderline, or malignant lesions. Benign and borderline PTs can grow permanently and recur if excised incompletely, an issue for malignant PTs as well [7,8]. Consequently, PTs diagnosed by CNB have been conventionally managed with surgical wide excision which enables the accurate diagnosis of the PT subtype and appropriate treatment [5,9]. Considering that many different therapeutic strategies exist, it is crucial to accurately diagnose FELs before surgery in clinical practice. However, conventional B-mode ultrasonography (US), widely-used to diagnose breast lesions, cannot sufficiently differentiate FAs from PTs due to overlapping imaging features such as oval shape, circumscribed margin, or hypoechogenicity [10,11]. While US-guided CNB (US-CNB) is considered to be the optimal method for preoperative diagnosis of PTs as well as most breast masses [12,13], CNB pathology is unable to distinguish all FELs into FAs and PTs, because CNB specimens only show part of the total lesion and histologic characteristics can overlap between FAs and PTs [1,5,14]. Thus, when CNB specimens show PT features but a definitive diagnosis cannot be made, the pathologist may diagnose a breast mass as a FEL with possibility for both FA and PT [6]. In this situation, patients might have to undergo unnecessary surgical excision just to confirm FA by surgical pathology.

US elastography (USE) and color Doppler US have been investigated as ancillary imaging tools used to improve diagnostic performances of conventional B-mode US for breast lesions. Among various USE techniques, shear-wave elastography (SWE) is highly reproducible and quantifies tissue stiffness in kilopascals (kPa) or meters per second [15]. Recent studies have reported that the addition of SWE to B-mode US improves its diagnostic performance in differentiating benign breast lesions from malignant breast lesions [15,16]. Additional use of Doppler US increased the specificity of B-mode US combined with mammography to 97.6% from 76.2% for diagnosing breast malignancies [17].

To our knowledge, the potential benefits of SWE and color Doppler US have not been established for differentiation of FA and PT, although previously, strain elastography techniques using freehand manual compression showed elastic differences between FA and PT [18,19] and hypervascularity on Doppler US was a significant feature of PT compared to FA [20]. Therefore, the purpose of this study was to evaluate SWE and color Doppler US features for FA and PT, and to determine the utility of combined SWE and color Doppler US for preoperative differentiation of FA and PT.

## Materials and methods

### Patients and lesions

This retrospective study was approved by the institutional review board of Samsung Medical Center, and informed consent was waived (2015-06-153). The data file is available in a public repository, DRYAD digital Repository (doi:10.5061/dryad.sh511).

**Fig 1:**
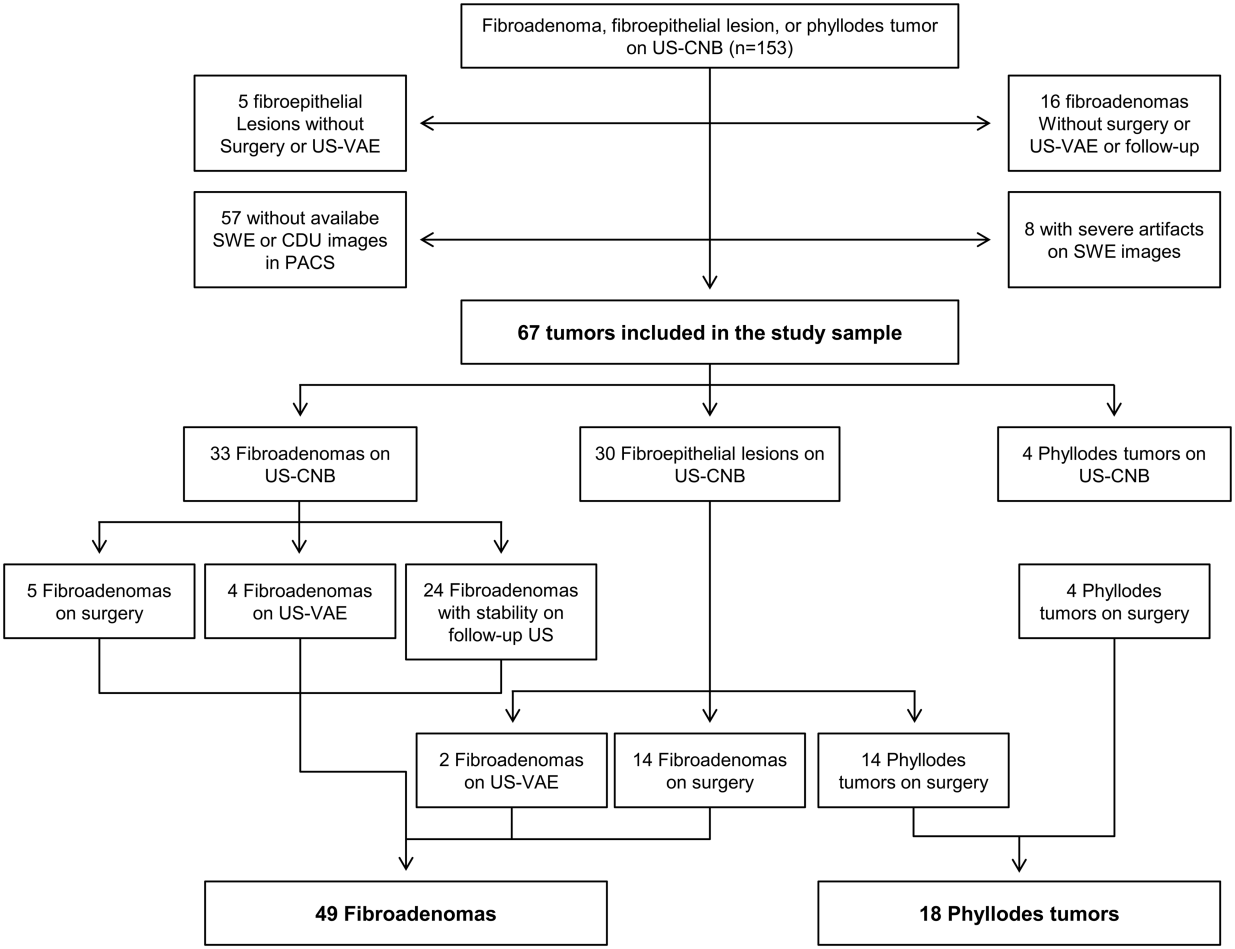
Flowchart of study sample selection. *US*- *CNB*, Ultrasonography-guided core needle biopsy; *US-VAE*, Ultrasonography-guided vacuum-assisted excision; *PACS*, Picture archiving and communications system; *SWE*, Shear-wave elastography.

From November 2013 to January 2015, 1328 women at average risk for breast cancer consecutively underwent US-CNB after diagnostic or screening breast US examinations using the Aixplorer system (SuperSonic Imagine, Aix en Provence, France) equipped with SWE. Women with a personal history of breast cancer surgery, high risk women with a family history of breast cancer or genetic carriers, or women who were previously diagnosed with high risk lesions were not included. The inclusion criteria were as follows: (a) women who were diagnosed with FA, FEL with a possibility of both FA and PT (equivocal FEL), or PT by US-CNB, (b) women with FELs or PTs diagnosed by US-CNB who underwent surgical or US-guided vacuum-assisted excision (US-VAE), (c) women whose FAs showed stability or decrease in size on follow-up US after more than 1 year, and (d) women who had B-mode US, SWE, and color Doppler US images available in the picture archiving and communications system [PACS] prior to US-CNB. According to the inclusion criteria, 153 lesions in 151 women, which were diagnosed as FA, equivocal FEL, or PT by US-CNB, were found for the study period. The exclusion criteria were then applied and were as follows: (a) women without subsequent surgical excision or US-VAE of their FEL or PT diagnosed by US-CNB, (b) women with FAs who did not undergo surgery but were either lost to follow-up after more than 1 year or showed increase in size on follow-up US, and (c) women without B-mode US, SWE or color Doppler US images available in the PACS. According to the above exclusion criteria, 86 lesions of 84 patients were excluded for the following reasons: 57 without available SWE or color Doppler US data; 8 with severe artifacts seen on SWE images [e.g. superficial lesions located less than 5 mm in depth, calcified lesions]; 16 FAs without subsequent surgery, US-VAE, or follow-up US for more than 1 year; and 5 equivocal FELs without subsequent surgery or US-VAE. Finally, excluding these 86 leisons of 84 patients from 153 lesions of 151 patients, 67 lesions of 67 patients were included in the study population (Fig 1).

US-CNB diagnoses of the 67 lesions were 33 FAs, 30 equivocal FELs, and 4 PTs. Nine of the 33 FAs on US-CNB were confirmed as FA through surgical excision (n = 5) or US-VAE (n = 4). The other 24 FAs were observed with follow-up US studies for more than 1 year. The mean period of follow-up US was 21.8 months (range, 20–26 months) and no interval change was observed on follow-up US studies. Of the 30 equivocal FELs on US-CNB, 28 underwent surgical excision (14 FAs, 11 benign PTs and 3 borderline PT) and 2 underwent US-VAE (2 FAs). All four PTs on US-CNB underwent surgical excision (3 benign PTs and 1 borderline PT).

### US examinations

US examinations were performed by one of 3 board-certified radiologists with exclusive experience in breast imaging for more than 9 years. The Aixplorer system (SuperSonic Imagine, Aix en Provence, France) equipped with a 15–4 MHz linear array transducer was used. The radiologists performed bilateral whole breast conventional B-mode US examinations and obtained at least two orthogonal (either transverse and longitudinal or radial and antiradial planes) B-mode US images for each breast lesion. Parameters for B-mode breast US were set as follows: dynamic range 68 dB, tissue tuner 1480 m/s, 2-dimensional gain 25%, frame rate 55Hz, and compound imaging in general. They subsequently measured lesion size (maximal diameter). In our institution, patients usually underwent both US and mammography in the diagnostic setting since most patients were referred to our institution, a tertiary referral center, after undergoing screening examinations at other outside hospitals. The radiologists determined the Breast Imaging Reporting and Data System (BI-RADS) final assessment category (1, 2, 3, 4a, 4b, 4c, or 5) independently based on B-mode US or mammography, then recommended further management according to the higher category of the two BI-RADS categories assigned through B-mode US and mammography [21]. BI-RADS category 3 was assigned to oval, circumscribed, isoechoic masses with parallel orientation [21]. BI-RADS category 4a, 4b, 4c or 5 was assigned according to the number of suspicious features [21,22]. Biopsies were performed on lesions assessed as equal to or more than category 4a assessment (n = 45), category 3 lesions with palpable mass (n = 4), or increase in size (n = 5) and category 3 lesions at patient-request (n = 13). After conventional B-mode US, targeted SWE and color Doppler US examinations were routinely performed for the lesion scheduled for US-CNB by the same radiologist. Two-orthogonal view SWE and color Doppler US images showing the most suspicious features were obtained. Customized settings of the SWE features were used, with the preference for the penetration mode in cases of poor penetration. Tissue elasticity was depicted with a color-coded map in kPa for each pixel with a color scale ranging from 0 (dark blue; soft) to 180 kPa (red; stiff). Tissue elasticity was quantified with a 2-mm circular region-of-interest (Q-box) drawn over the stiffest portion of the lesion including the immediately adjacent stiffest tissue [15,23–25]. Quantitative elasticity values including the mean elasticity (E_mean_) and maximal elasticity (E_max_) were automatically calculated. A standard equipment setting for breast imaging was used for coor Doppler US. Doppler amplification was set to a level in which normal breast parenchyma did not display any noise and which was just under the level in which random noise appeared. The settings were as follows: medium wall filter, pulse repetition frequency 700 Hz, intermediate persistence, frame rate of 16 frames per second and a dynamic range of 50 dB.

US-CNB was performed for at least four passes using a 14-gauge automated biopsy gun (Acecut, TSK Laboratory, Soja, Japan) or a 14-gauge dual-action semiautomatic core biopsy needle with a 22-mm throw (Stericut cut with coaxial; TSK laboratory, Tochigi, Japan). US-VAE was performed with an 11-gauge or 8-gauge needle (Mammotome; Devicor Medical, Cincinnati, OH, USA).

### Image analysis

The B-mode US, SWE, and color Doppler US images in the PACS were retrospectively reviewed by two radiologists (K.G.R. and B.K.H.) in consensus. The radiologists were blinded to any information regarding patient history, findings with other imaging modalities such as mammography, or final diagnosis. B-mode US features were assessed according to shape (oval, round, or irregular), orientation (parallel or not parallel), margin (circumscribed, or not circumscribed [indistinct, angular, microlobulated, or spiculated]), echo pattern (anechoic, hyperechoic, complex cystic and solid, hypoechoic, isoechoic, or heterogeneous), and posterior features (no posterior features, enhancement, shadowing, or combined pattern) based on BI-RADS. Final assessment equal to or higher than BI-RADS category 4 was made when the lesion had 1 or more suspicious US features [21,22]. The values of E_mean_ and E_max_ were acquired from the targeted SWE images. Vascularity on color Doppler US images was scored according to the number of vessels which were depicted within, surrounding, or penetrating the lesions [26] and classified into two categories: low (when no flow or one vessel-flow signal was observed) and high (when equal to or greater than 2 vessel-flow signals were observed).

Among 67 patients, 39 underwent concurrent diagnostic mammographies with their US examinations; the other 28 patients who were younger than 40 years (n = 20) or who had undergone mammographies within the previous year (n = 8) did not undergo concurrent mammographies. Two radiologists who did not participate in the US image review retrospectively assessed the mammographies to correlate the mammographic and US findings of the lesion. In addition, they evaluated mammographic breast density according to BI-RADS [21], and classified breast density as ‘non-dense’ (almost entirely fatty, scattered areas of fibroglandular density) or ‘dense’ (heterogeneously dense, extremely dense).

### Histopathologic analysis

Histopathological features of US-CNB, US-VAE, and surgical excision specimens were assessed for stromal characteristics including cellularity, atypia, mitosis, tissue fragmentation, and tumor margin [1]. US-CNB results were classified as FA, equivocal FEL, or PT. Equivocal FELs were diagnosed when CNB specimens showed PT features that were not definitive. US-VAE and surgical excision results were classified into FA or PT.

### Data and statistical analyses

Final diagnosis was based on histopathological results. PTs were confirmed with surgery or US-VAE. FAs were confirmed with (a) surgery or US-VAE or (b) US-CNB and no interval change or decreased size on follow-up US for more than 1 year. Concordance or discordance of pathologic diagnoses between US-CNB and excision biopsy results (surgery or US-VAE) was assessed for 43 lesions for which both US-CNB and excision biopsy had been performed.

Age, lesion size on B-mode US, SWE features (E_mean_ and E_max)_ and vascularity on the color Doppler US of FAs and PTs were compared between PT and FA groups. All numeric data were checked for normal distribution by the Kolmogorov-Smimov goodness-of-fit test. The Mann-Whitney U test was performed since the data were not normally distributed. Non-numeric data were analyzed using the chi-square test or Fisher's exact test. In the analysis of diagnostic performance for differentiating PTs from FAs, positive test results for PT were defined as BI-RADS category 4a or higher for B-mode US and high vascularity (equal to or greater than 2 vessel-flow signals) for color Doppler US, respectively. In terms of SWE features, positive results of quantitative elasticity values (E_mean_ and E_max_) were determined using the cut-off values obtained according to the Youden index [27]. The sensitivities, specificities, accuracies, positive predictive values (PPVs), and negative predictive values (NPVs) of B-mode US, SWE features (E_mean_ and E_max_) and color Doppler US were calculated to differentiate PTs from FAs. The area under the curve (AUC) was also calculated after construction of the receiver operating characteristic (ROC) curve. The AUC, sensitivity, specificity, accuracy, PPV, and NPV of B-mode US alone were compared with those of SWE, color Doppler US or the combination of SWE and color Doppler US, respectively with McNemar’s test, the chi-square test and Bennett’s test [28]. Post-hoc analysis for multiple comparison was performed using the Bonferroni correction and adjusted *P* values were provided.

For the subgroup of 30 equivocal FELs diagnosed by US-CNB, the diagnostic performances of B-mode alone and the combined use of SWE and color Doppler US were also evaluated.

Analysis was performed using SAS version 9.4 (SAS Institute, Cary, NC) and R 3.2.2 (Vienna, Austria; http://www.R-project.org/). Statistical significance was accepted when *P* values < 0.05.

## Results

The median age of the 67 patients was 42.0 years (interquatile range [IQR], 37.0–45.0 years). The median lesion size of the 67 lesions measured by US was 14.0 mm (IQR, 11.0–20.0). Of the 67 lesions, there were 49 FAs (73.1%) and 18 PTs (26.9%). Among 43 lesions for which both US-CNB and excision biopsy were performed, FAs (n = 9) and PTs (n = 4) on CNB pathology were all diagnosed concordantly on surgical or US-VAE specimens. Of the 30 equivocal FELs diagnosed with CNB, 16 were confirmed as FAs (53.3%) and 14 as PTs (46.7%) by surgery or US-VAE (Fig 1).

**Fig 2:**
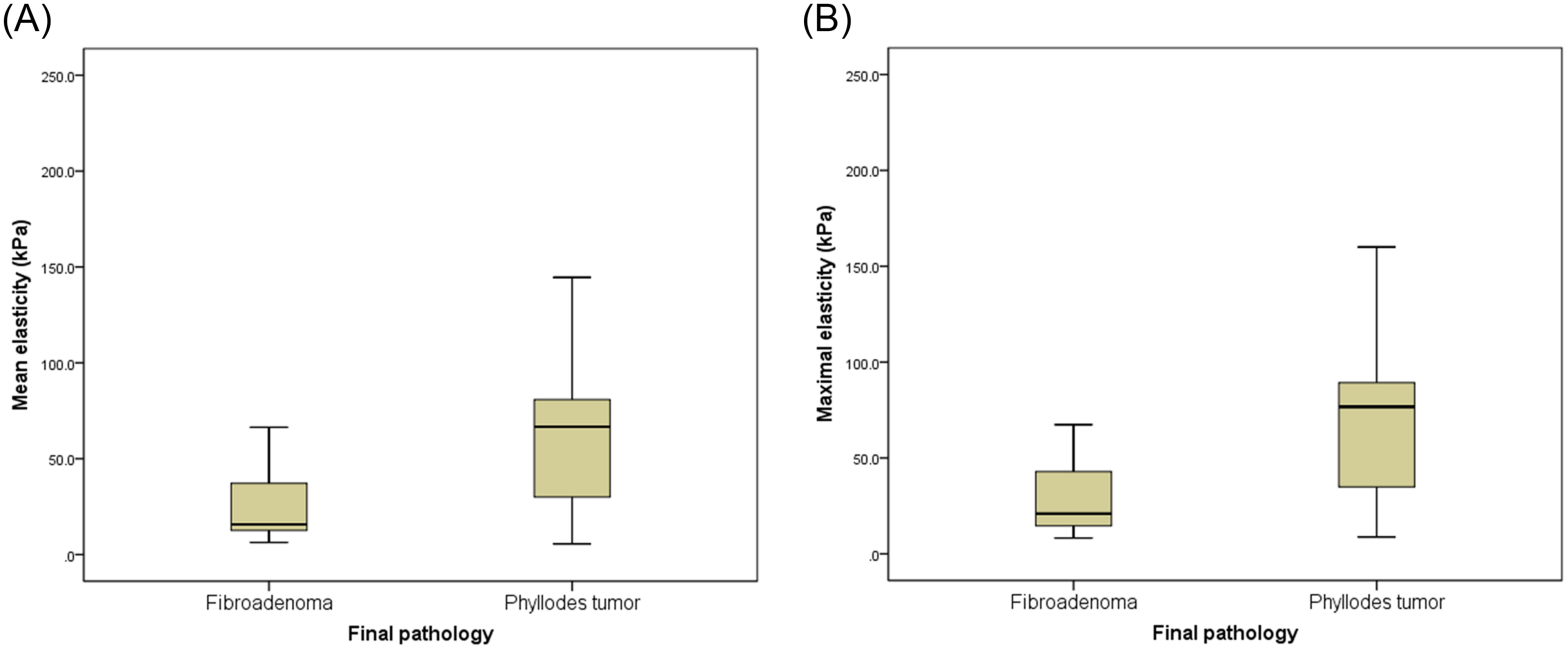
Box plots of tissue stiffness by shear-wave elastography. Both E_mean_ (mean elasticity) (a) and E_max_ (maximal elasticity) (b) were significantly higher in phyllodes tumor than fibroadenoma.

The clinical and imaging characteristics of the FA and PT groups are shown in Table 1. The median lesion size of PTs was significantly larger than FAs (26.0 mm vs. 13.0 mm, *P*<0.01). Of the B-mode US features, the heterogeneous echo pattern was more frequently observed with significance in PTs than FAs (61.1% vs. 24.5%, *P*<0.01). The other B-mode US features were not significantly different between two groups. BI-RADS categories based on B-mode US were higher in PTs than FAs (*P*< 0.01). Both E_mean_ and E_max_ were significantly higher in PTs than FAs (E_mean_, 66.7 vs. 15.7 kPa, *P* < 0.01; E_max_, 76.7 vs. 21.0 kPa, *P* <0.01) (Fig 2). On the other hand, low vascularity (no vascularity or one vessel) on color Doppler US were more frequent in FAs than in PTs (*P* < 0.01). Of 39 patients who underwent concurrent mammographies, 3 (7.7%) had non-dense breasts and the other 36 (92.3%) had dense breasts. Among these 39 patients, 20 had no mammographic findings correlating to their US findings whereas the other 19 lesions of 19 patients presented as ‘mass’ (n = 18) and ‘focal asymmetry’ (n = 1) on mammography.

**Table 1:** Characteristics of the 67 breast fibroepithelial lesions in 67 patients.

	Fibroadenoma (n = 49)	Phyllodes tumor (n = 18)	*P*
Age (years)	42.0 (37.5–45.5)	38.5 (31.0–43.5)	0.06
B-mode US			
Lesion size (mm)	13.0 (10.0–16.5)	26.0 (14.0–34.5)	<0.01
BI-RADS Lexicon			
Shape			0.42
Oval	38 (77.6)	15 (83.3)	
Round	1 (2.0)	1 (5.6)	
Irregular	10 (20.4)	2 (11.1)	
Orientation			0.31
Parallel	44 (89.8)	18 (100.0)	
Not parallel	5 (10.2)	0 (0.0)	
Margin			0.78
Circumscribed	29 (59.2)	12 (66.7)	
Not circumscribed	20 (40.8)	6 (33.3)	
Echo pattern			<0.01
Isoechoic	29 (59.2)	2 (11.1)	
Hypoechoic	8 (16.3)	5 (27.8)	
Heterogeneous	12 (24.5)	11 (61.1)	
Posterior features			0.12
No posterior features	47(95.9)	15 (83.3)	
Enhancement	0 (0.0)	0 (0.0)	
Shadowing	2 (4.1)	3 (16.7)	
BI-RADS Category			<0.01
3	21 (42.9)	1 (5.6)	
4A	24 (49.0)	15 (83.3)	
4B	4 (8.2)	2 (11.1)	
Shear-wave elastography			
Mean elasticity (E_mean_, kPa)	15.8 (12.6–38.2)	66.7 (28.8–83.9)	<0.01
Maximum elasticity (E_max_, kPa)	21.0 (14.6–44.4)	76.7 (34.8–93.2)	<0.01
Color Doppler US			<0.01
No vessel	20 (40.8)	0 (0.0)	
One vessel	11 (22.4)	1 (5.6)	
Equal to or greater than 2 vessels	18 (36.7)	17 (94.4)	

*P* values indicate comparisons between the fibroadenoma group and phyllodes tumor group. Numeric data are presented as median value (interquatile range). Non-numeric data are presented as the number of lesions (percentage). BI-RADS, Breast Imaging Reporting and Data System.

The diagnostic performances of B-mode US, SWE features and color Doppler US for differentiating PT from FA are summarized in Table 2. The AUCs of color Doppler US (0.789) and SWE features found with cut-off values of E_mean_ 43.9 kPa (0.782) and E_max_ 46.1 kPa (0.759) were higher than the AUC of B-mode US (0.687), without statistical significance. E_mean_ showed significantly higher specificity (89.8%) and accuracy (83.6%) than those of B-mode US (specificity 42.9%, accuracy 56.7%) (P<0.01). E_max_ showed significantly higher specificity (79.6%) than B-mode US (P<0.01). However, the sensitivities of E_mean_ (66.7%) and E_max_ (72.2%) were lower than B-mode US (94.4%) (P>0.05). Color Doppler US had the same sensitivity but higher values for the other diagnostic performance values compared to B-mode US, without statistical significance (P>0.05).

**Table 2:** Diagnostic performances of B-mode US, SWE and color Doppler US in differentiating phyllodes tumor from fibroadenoma.

	AUC	Sensitivity	Specificity	Accuracy	PPV	NPV
B-mode US						
BI-RADS category ≥4A	0.687	94.4% (17/18)	42.9% (21/49)	56.7% (38/67)	37.8% (17/45)	95.5% (21/22)
SWE						
E_mean_ >43.9 kPa*	0.782	66.7% (12/18)	89.8% (44/49)	83.6% (56/67)	70.6% (12/17)	88.0% (44/50)
*P* value	0.76	0.37	<0.01	<0.01	0.07	0.77
E_max_ >46.1kPa*	0.759	72.2% (13/18)	79.6% (39/49)	77.6% (52/67)	56.5% (13/23)	88.6% (39/44)
*P* value	>0.99	0.67	<0.01	>0.99	0.19	0.92
Color Doppler US						
High vascularity†	0.789	94.4% (17/18)	63.3% (31/49)	71.6% (48/67)	48.6% (17/35)	96.9% (31/32)
*P* value	0.43	>0.99	0.22	0.53	0.33	>0.99
Combination of SWE and color Doppler US						
E_mean_ >43.9 kPa or high vascularity	0.786	100% (18/18)	57.1% (28/49)	68.7% (46/67)	46.2% (18/39)	100% (28/28)
*P* value	0.28	>0.99	0.84	>0.99	0.46	>0.99
E_max_ >46.1kPa* or high vascularity	0.755	100% (18/18)	51.0% (25/49)	64.2% (43/67)	42.9% (18/42)	100% (25/25)
*P* value	0.91	>0.99	>0.99	>0.99	>0.99	>0.99

Data are percentages with the number of phyllodes tumors to the number of lesions in parentheses. *P* values indicate comparisons between B-mode US and SWE or between B-mode US and color Doppler US. SWE, shear-wave elastography; AUC, area under the curve; E_mean_, mean elasticity (kPa); E_max_, maximum elasticity (kPa); PPV, positive predictive value; NPV, negative predictive value.

*Cut-off values for E_mean_ and E_max_ were set based on the Youden index.AUC, area under the curve.

^†^Vascularity on color Doppler US images was classified into low (no flow or only one vessel signal was observed) and high (equal to or greater than 2 vessel-flow signals were observed).

**Fig 3:**
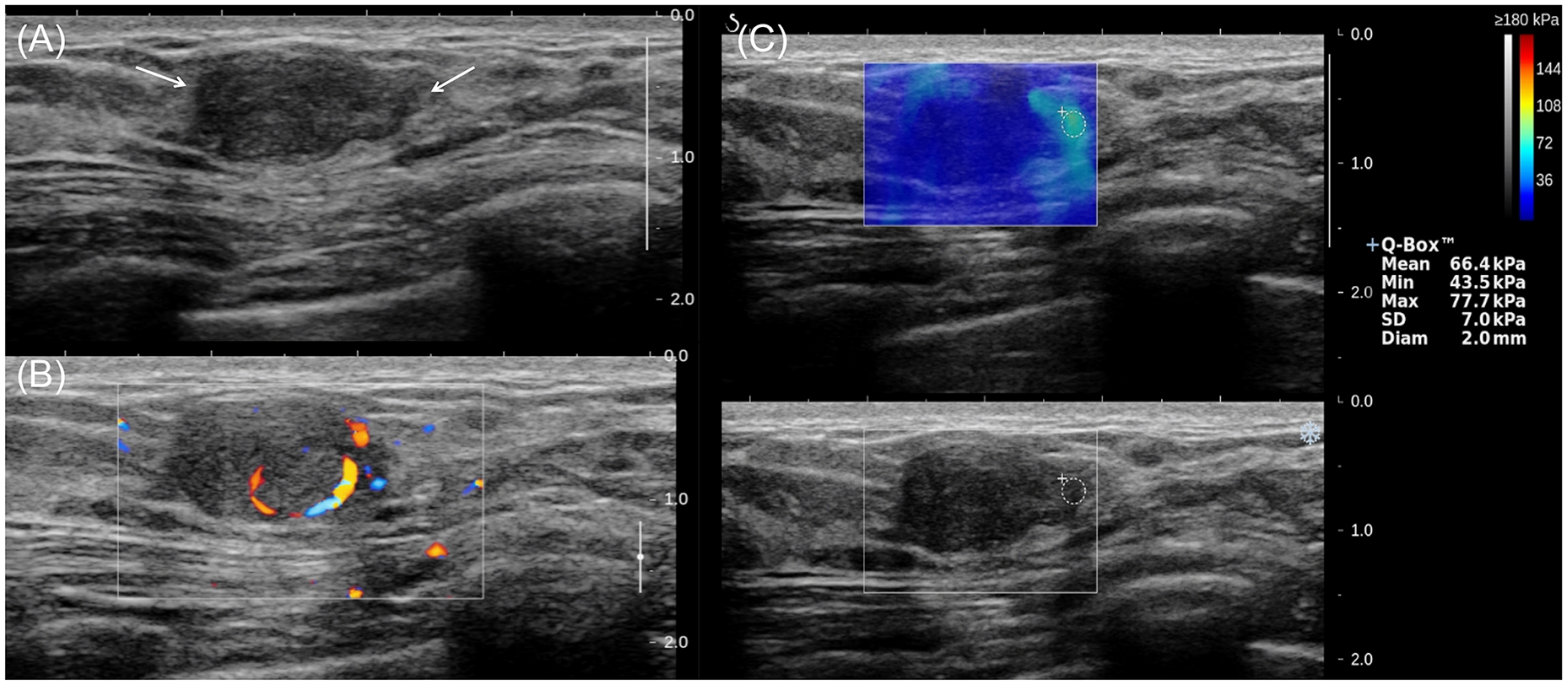
A 45-year-old woman diagnosed with phyllodes tumor by US-guided core needle biopsy, which was confirmed as benign phyllodes tumor on surgical excision. (a) The B-mode US image shows a 1.5-cm breast mass assessed as BI-RADS category 4a (arrows), (b) the color Doppler US image shows high vascularity, and (c) the shear-wave elastography image shows E_mean_ of 66.4kPa and E_max_ of 77.7kPa.

**Fig 4:**
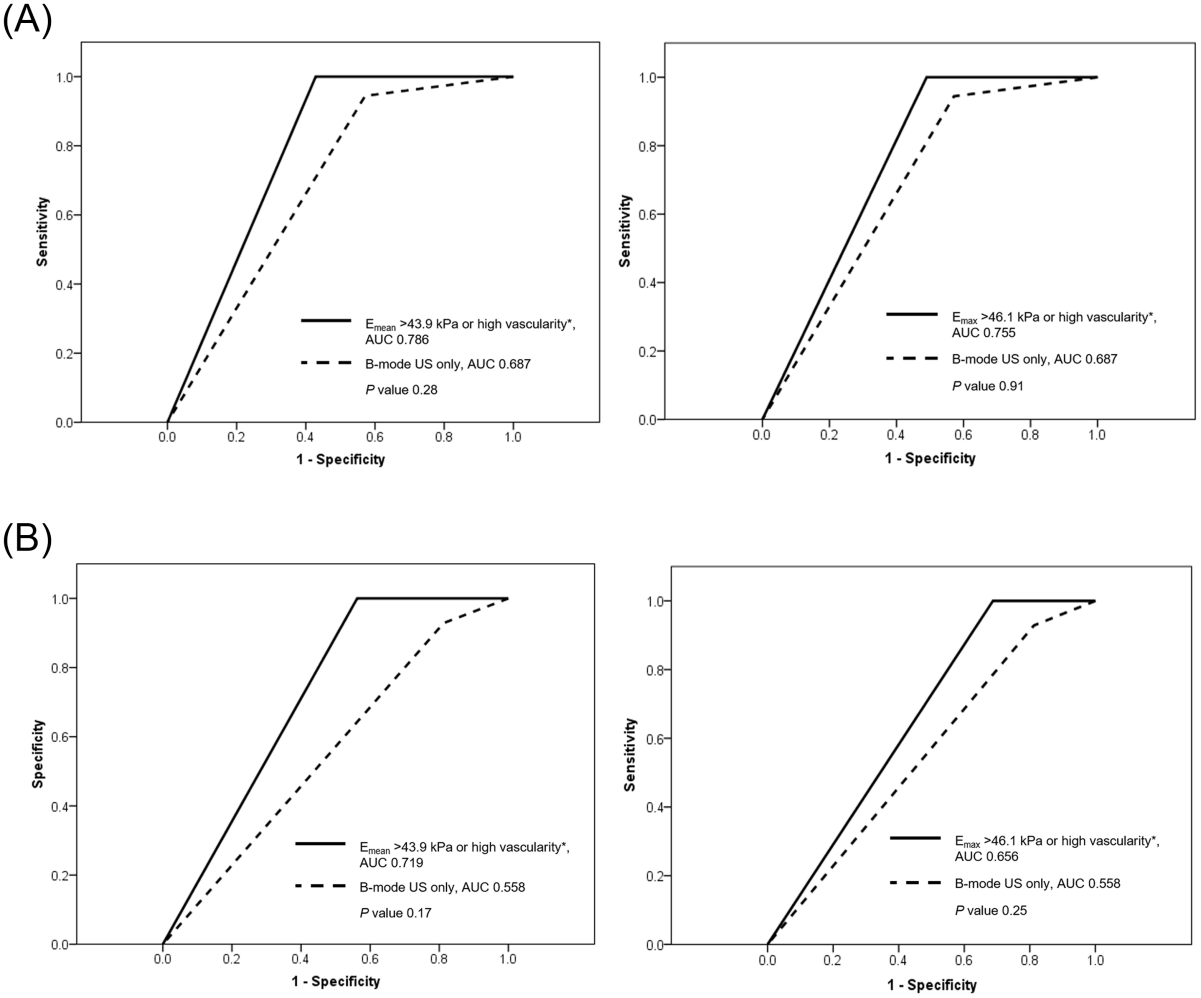
Receiver operating characteristic curves of B-mode US alone and combined use of shear-wave elastography (SWE) and color Doppler US. (a) For the total 67 fibroepithelial lesions, the area under the curves (AUCs) for ‘E_mean_ >43.9 kPa or high vascularity*’ (0.786, left) and ‘E_max_ > 46.1 kPa or high vascularity*’ (0.755, right), were compared to the AUC of B-mode US (0.687). (b) For the subgroup of 30 equivocal fibroepithelial lesions, AUCs for ‘E_mean_ >43.9 kPa or high vascularity*’ (0.719, left) and ‘E_max_ > 46.1 kPa or high vascularity*’ (0.656, right) were compared to the AUC of B-mode US (0.558). *Equal to or greater than 2 vessel-flow signals were observed on color Doppler US.

**Fig 5:**
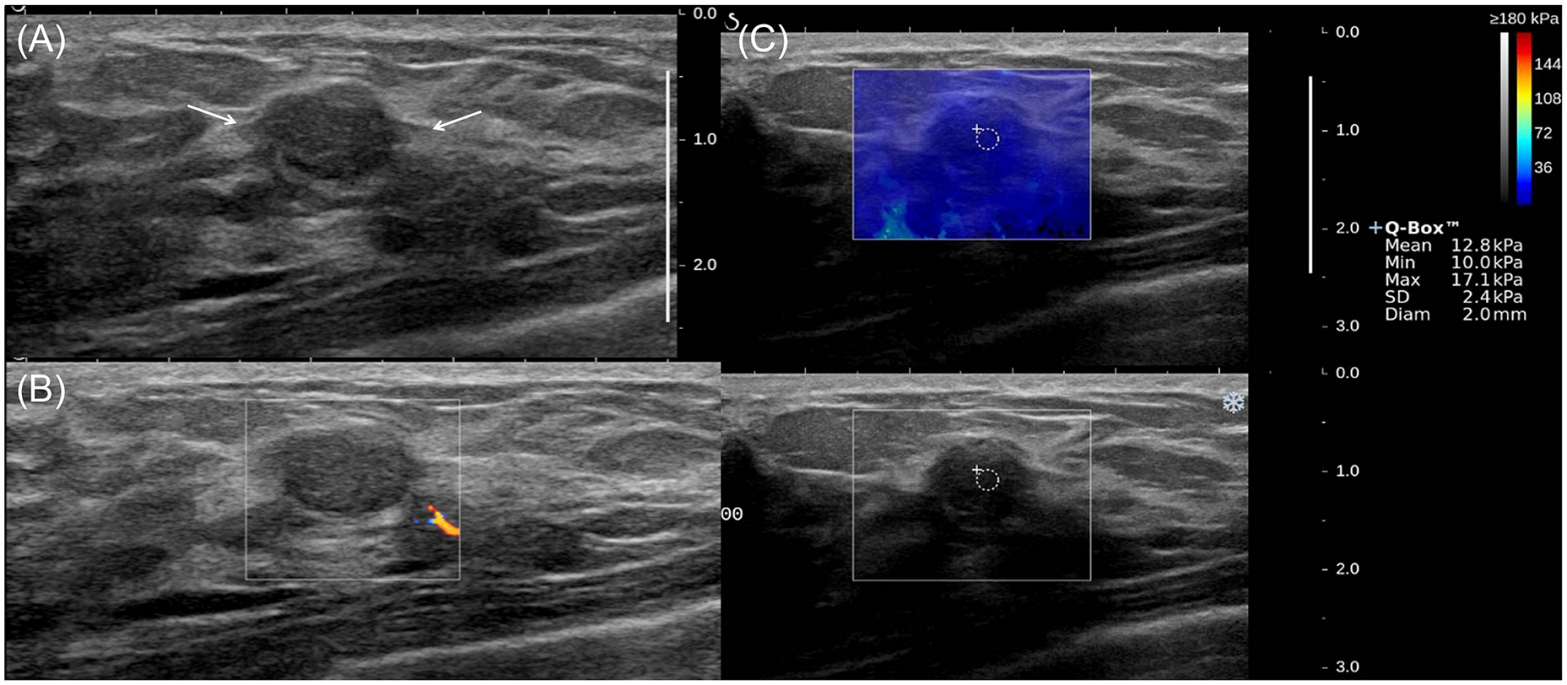
A 36-year-old woman diagnosed with equivocal fibroepithelial lesion by US-guided biopsy, which was confirmed as fibroadenoma on surgical excision. (a) The B-mode US image shows a 1.4-cm breast mass with BI-RADS category 4a (arrows), (b) the color Doppler US image shows low vascularity, and (c) the SWE image shows E_mean_ of 12.8 kPa and E_max_ of 17.1 kPa.

The combination of SWE and color Doppler US with the criterion ‘‘E_mean_ > 43.9 kPa or high vascularity’ or ‘E_max_ >46.1 kPa or high vascularity’ improved both sensitivity and NPV to 100% compared to SWE or color Doppler US alone (Table 2 and Fig 3). The two combined modes of SWE and color Doppler US using E_mean_ and E_max_ showed a larger AUC and higher sensitivity, specificity, accuracy, PPV, and NPV compared to those of B-mode US alone without statistical significance (Table 2 and Fig 4a). For the subgroup of 30 equivocal FELs on CNB pathology, the diagnostic performance of the combined use of SWE and color Doppler US using the criterion ‘E_mean_ > 43.9 kPa or high vascularity’ or ‘E_max_ >46.1 kPa or high vascularity’ was evaluated (Table 3 and Fig 4b). The combined use of SWE and Doppler US using E_mean_ and E_max_ showed larger AUCs and higher diagnostic values compared to those of B-mode US, without statistical significance (P>0.05), in differentiating PTs from FAs for the equivocal FEL subgroup. Of the 30 equivocal FELs, all of the 7 lesions (23.3%, 7/30) that simultaneously showed E_mean_ ≤ 43.9 kPa and low vascularity were finally confirmed as FAs by excision biopsy (Fig 5).

**Table 3:** Diagnostic performances of B-mode US and combined SWE and color Doppler US in differentiating phyllodes tumor from fibroadenoma for the subgroup of 30 equivocal fibroepithelial lesions diagnosed by core-needle biopsy.

	B-mode US	Combination of SWE and color Doppler US			
	BI-RADS category ≥4A	E_mean_ >43.9 kPa or high vascularity*	*P*	E_max_ > 46.1 kPa or high vascularity*	*P*
AUC	0.558	0.719	0.17	0.656	0.25
Sensitivity	92.9% (13/14)	100% (14/14)	>0.99	100% (14/14)	>0.99
Specificity	18.8% (3/16)	43.8% (7/16)	0.58	31.3% (5/16)	>0.99
Accuracy	53.3% (16/30)	70.0% (21/30)	0.58	63.3% (19/30)	>0.99
PPV	50.0% (13/26)	60.9% (14/23)	0.27	56.0% (14/25_	0.58
NPV	75.0% (3/4)	100% (7/7)	0.70	100% (5/5)	0.72

Data are percentages with the number of phyllodes tumors or the number of lesions in parentheses. *P* values indicate comparisons between B-mode US and the combination of SWE and color Doppler US. SWE, shear-wave elastography; BI-RADS, Breast Imaging Reporting and Data System; AUC, area under the curve; E_mean_, mean elasticity (kPa); E_max_, maximum elasticity (kPa); PPV, positive predictive value; NPV, negative predictive value.

*Vascularity on color Doppler US images was classified into low (no flow or only one vessel signal was observed) and high (equal to or greater than 2 vessel-flow signals were observed).

## Discussion

In this study, FAs showed significantly lower values of SWE quantitative features (E_mean_ and E_max_) than those of PTs, which indicates that FAs may have less stiffness than PTs. These results are in accordance with the results of a previous study using strain elastography [19]. PTs are histologically characterized with more abundant and cellular stroma than FAs [1,5]. Accordingly, PTs tend to be stiffer or firmer than FAs. PT and FA are biphasic neoplasms composed of a proliferation of epithelial and stromal components [29,30] and consequently, the histologic characteristics of these lesions can overlap [29–31]. This might be the reason behind the substantial overlap between the E_mean_ and E_max_ values of the PT and FA groups (Fig 2), which led to the false-positive or false-negative results of SWE in our study. Other studies have also assessed the color Doppler US features of PT and FA [32,33]. These studies used variable features (e.g. resistance index, pulsatility index, or systolic peak flow velocity), but they could not demonstrate the utility of color Doppler US in the differential diagnosis of PT and FA. We used a simple scoring system based on the number of vessels observed on color Doppler US, a method which may be easily applied to clinical practice; we found that FAs showed significantly lower vascularity (no flow or one vessel) than PTs.

To diagnose FELs, a heterogeneous echo pattern and presence of round cysts/clefts within a mass have been suggested as conventional B-mode US features that could help distinguish PTs from FAs [10,11,34]. Similarly to previous studies, the heterogeneous echo pattern was a significantly frequent B-mode US feature in PTs, which might reflect their stromal heterogeneity or internal cystic structures [10,34,35]. However, the heterogeneous echo pattern cannot be regarded as specific or pathognomonic for PTs, because it can be observed in a substantial number of FAs (13.5–51%) [10,34]. On the other hand, the other B-mode US features according to the BI-RADS lexicons for 'mass' were not different between PTs and FAs in prior studies as well as our study [10,34]. Considering the results of previous studies along with our own results, conventional B-mode US may have limited value in differentiating PTs and FAs.

In terms of diagnostic performance for distinguishing PTs from FAs, all SWE features showed higher specifities (79.6–89.8%) for PTs compared to B-mode US (42.9%) and color Doppler US (63.3%). Meanwhile, both color Doppler US and B-mode US showed higher sensitivities (94.4%, respectively) than the SWE features (66.7–72.2%). The combined use of SWE and color Doppler US showed relatively higher diagnostic performances, compared to those of B-mode US alone. However, the PPV of the combined use of SWE and color Doppler US (46.2%) was still not high. These results indicate that the additional use of SWE and color Doppler US may not be able to replace US-CNB as the main diagnostic tool when differentiating FAs and PTs.

For the 43 lesions for which both US-CNB and excision biopsy were performed, we assessed the diagnostic concordance or discordance between US-CNB and excision biopsy. Our results demonstrated that all of the FAs (n = 9) and PTs (n = 4) on CNB pathology were diagnosed concordantly on surgical or US-VAE specimens. This finding reflects that FELs diagnosed as FA or PT by US-CNB can be properly managed on the assumption of concordance between US-CNB and excision biopsy [1,5,6]. On the other hand, all 30 equivocal FELs diagnosed by US-CNB in this study underwent subsequent surgery or US-VAE. Among these, 14 lesions (46.7%) were upgraded to PTs by excision biopsy. The other 16 equivocal FELs (53.3%) were confirmed as FAs by excision biopsy and these 16 patients with equivocal FELs might be regarded as having undergone unnecessary further excision. Based on these results, we hypothesized that the combined use of SWE and color Doppler US may help clinicians preoperatively distinguish FAs from equivocal FELs diagnosed by US-CNB, considering that its NPV was 100%, and thus, may reduce the number of unnecessary diagnostic excision procedures. When a criterion of ‘E_mean_ ≤ 43.9 kPa and low vascularity’ was used to distinguish FAs from equivocal FELs, 23.3% (7/30) of equivocal FELs could have been diagnosed as FAs. Our results indicate that when diagnosing FELs, additional information about the elasticity and vascularity of the whole lesion volume may supplement the shortcomings of US-CNB which only provides information about the targeted portions of the lesion. Accordingly, the combined use of SWE and color Doppler US may help decide the best management approach for equivocal FELs diagnosed by US-CNB, and a follow-up without excision may be considered for equivocal FELs with ‘E_mean_ ≤ 43.9 kPa and low vascularity’.

There were several limitations in our study. First, we could not include all patients who had been diagnosed with FA or PT during the study period; thus, a selection bias might exist. Since this was a retrospective study using previously static-captured images, lesions which did not have available SWE and color Doppler US data were excluded. Second, this study had a relatively small sample population, and a further study with a large sample size is needed. Third, not all tumors were confirmed through total excision and a 1-year follow-up period for FAs without excision is too short to establish benignity. However, all 24 FAs without total excision were image-pathology concordant and showed stability during each follow-up period (range, 20–26 months). Accordingly, total excision might not affect the results of this study. If this is a limitation, it may be an inevitable one with this retrospective study design, because breast tumors with benign CNB results are usually managed with follow-up in clinical practice. Finally, SWE and color Doppler US in this study were performed by three radiologists; and interobserver variability may exist.

In conclusion, SWE and color Doppler US characteristics were significantly different between FA and PT. FAs have a tendency to have less stiffness and lower vascularity than PTs. The combination of SWE and color Doppler US may help patients with equivocal FELs diagnosed by CNB avoid unnecessary excision biopsies since all equivocal FELs diagnosed by CNB showing both softness and low vascularity in our study were confirmed as FAs.
